# Polyhexamethyleneguanidine Phosphate-Induced Cytotoxicity in Liver Cells Is Alleviated by Tauroursodeoxycholic Acid (TUDCA) via a Reduction in Endoplasmic Reticulum Stress

**DOI:** 10.3390/cells8091023

**Published:** 2019-09-03

**Authors:** Sou Hyun Kim, Doyoung Kwon, Seunghyun Lee, Sung Hwan Ki, Hye Gwang Jeong, Jin Tae Hong, Yun-Hee Lee, Young-Suk Jung

**Affiliations:** 1 Lab of Molecular Toxicology, College of Pharmacy, Pusan National University, Busan 46241, Korea (S.H.K.) (S.L.); 2 Department of Cellular and Molecular Pharmacology, University of California San Francisco, San Francisco, CA 94158-2280, USA; 3 College of Pharmacy, Chosun University, Gwangju 61452, Korea; 4 College of Pharmacy, Chungnam National University, Daejeon 34134, Korea; 5 College of Pharmacy, Chungbuk National University, Cheongju 28160, Korea; 6 College of Pharmacy and Research Institute of Pharmaceutical Sciences, Seoul National University, Seoul 08826, Korea

**Keywords:** ER stress, PHMG-P, cytotoxicity, liver, tauroursodeoxycholic acid

## Abstract

Polyhexamethyleneguanidine phosphate (PHMG-P) is a widely used polymeric antimicrobial agent known to induce significant pulmonary toxicity. Several studies have reported that the liver also can be a target organ of polyhexamethyleneguanidine (PHMG) toxicity, but the exact effect of this compound on liver cells is not well understood. To identify the mechanism of PHMG hepatotoxicity, HepG2 cells were exposed to PHMG-P for 72 h. The cell viability was significantly decreased by PHMG-P in a time- and concentration-dependent manner. The mitochondrial membrane potential was markedly reduced by PHMG-P and the apoptotic signaling cascade was activated. The increases observed in C/EBP homologous protein (CHOP), p-IRE, and p-JNK levels in PHMG-P-treated cells indicated the induction of endoplasmic reticulum stress. To verify the role of ER stress in PHMG-P-induced cytotoxicity, HepG2 cells were pretreated with the chemical chaperone, tauroursodeoxycholic acid (TUDCA) and then co-treated with TUDCA and PHMG-P for 24 h. Interestingly, TUDCA inhibited PHMG-P-induced ER stress and cytotoxicity in a dose-dependent manner. The apoptotic cell death and mitochondrial depolarization were also prevented by TUDCA. The proteins involved in the apoptotic pathway were all normalized to their control levels in TUDCA-treated cells. In conclusion, the results suggest that PHMG-P induced significant cytotoxicity in liver cells and ER stress-mediated apoptosis, which may be an important mechanism mediating this hepatotoxicity.

## 1. Introduction

Polyhexamethyleneguanidine (PHMG) is a polymeric antimicrobial agent belonging to the guanidine class of chemicals [1,2,3,4]. Two forms of its salt, PHMG phosphate (PHMG-P) and PHMG hydrochloride (PHMG-H), are widely used in fabric softeners, paints, detergents, and swimming pools, due to their potent bactericidal and fungicidal activities [5,6,7,8]. The main antimicrobial mechanism of guanidine derivatives is cell membrane disruption. Their cationic nitrogen interacts with negatively charged cell surface phospholipids, especially phosphatidylglycerol, which is a major component of the bacterial cell wall, eventually leading to the destruction of the membrane structure [6,9,10,11,12].

In contrast to its effect on microorganisms, PHMG is considered to be relatively safe in humans, as the guanidine groups cannot bind well to neutral phospholipids, such as phosphatidyl choline and phosphatidyl ethanolamine, in mammalian cell membranes [10,11]. Recently, however, significant toxicity of PHMG has been reported in mammals. In South Korea, PHMG-P was used as a water tank disinfectant in humidifiers until 2011. More than 258 cases of fibrotic lung disease and associated fatalities have been reported in patients exposed to mist containing PHMG-P [1,13]. Animal studies have also demonstrated the pulmonary toxicity of PHMG. The intratracheal instillation of PHMG-P (0.3–1.5 mg/kg) caused an elevation in proinflammatory cytokine levels, immune cell recruitment, collagen deposition, and fibroblast proliferation in the lungs of mice [14,15]. The rats exposed to PHMG-P (1.51 mg/m^3^) aerosol particles for 3 weeks also showed significant inflammatory and fibrotic responses in the lungs [4].

It has also been reported that the liver can be adversely affected by PHMG. In Russia, more than 12,500 patients who consumed illegally manufactured vodka containing PHMG-H (0.10–0.14%) were hospitalized due to acute cholestatic hepatitis [16,17]. In rats, acute oral administration of 600 mg/kg PHMG-H has been shown to increase serum alkaline phosphatase levels by 176.7%, and sub-chronic (90 days) oral gavage of this compound (0.006–0.036 mg/kg) causes mild pericentral hepatocyte degeneration [18]. Zebrafish exposed to PHMG-P concentrations up to 0.3% show elevated alanine aminotransferase and aspartate aminotransferase levels and fat accumulation in the liver [19]. Moreover, the inhalation or intratracheal instillation of radio-labeled PHMG-P results in increased tissue distribution. Further, the persistence of this compound in the liver compared to other tissues, such as the heart, kidney, spleen, and thyroid in rodents [13], suggests that the liver may also be an important target of PHMG-induced toxicity.

In mammalian cells, the destruction of the plasma membrane has been suggested to be the main reason for PHMG cytotoxicity [2,3]. Recently, however, several groups have reported that apoptosis is an important mechanism of PHMG-induced pulmonary cell injury. The incubation of human bronchial epithelial (BEAS-2B) cells with PHMG-P at 16.0 µg/mL for 6 h, resulted in 40% lethality, and approximately 80% of the dying cells were found to be in the late phase of apoptosis [2]. Moreover, the genes related to apoptosis are significantly induced in human alveolar epithelial (A549) cells treated with PHMG-H [20], indicating that PHMG can cause apoptotic cell death.

In contrast to pulmonary cells, the exact effect of PHMG on liver cells has not yet been elucidated. Therefore, the purpose of this study was to evaluate the hepatotoxicity of PHMG and identify the cellular mechanism involved. The human liver-derived HepG2 cells were exposed to PHMG-P and the toxic responses, particularly apoptotic cell death and its related signaling pathways, were monitored. Moreover, to investigate the association between ER stress and PHMG-induced cellular injury, tauroursodeoxycholic acid (TUDCA), which is known to inhibit ER stress, was used as a pre- and co-treatment with PHMG-P. PHMG-P was shown to induce significant levels of apoptosis and ER stress, and the inhibition of ER stress by TUDCA prevented this apoptotic cell death.

## 2. Materials and Methods

### 2.1. Cell Culture

The HepG2 human liver cells (ATCC, Manassas, VA, USA) were maintained in Dulbecco’s modified Eagle’s medium (DMEM; Hyclone, Logan, UT, USA) supplemented with 10% fetal bovine serum (FBS, Hyclone), 4 mM glutamine (Hyclone), 100 U/mL penicillin (Hyclone), and 100 μg/mL streptomycin (Hyclone). The AML12 mouse liver cells (ATCC) were grown in DMEM/F-12 medium (Hyclone) supplemented with 10% FBS, 2.5 mM glutamine, 15 mM HEPES, 100 U/mL penicillin, and 100 μg/mL streptomycin. The medium was supplemented with 1X Insulin-Transferrin-Selenium-G (Gibco, Life Technologies™, New York, NY, USA), and 40 ng/mL dexamethasone (Sigma-Aldrich, St. Louis, MO, USA). The cells were incubated at 37 °C in a humidified atmosphere and 5% CO_2_.

### 2.2. Cell Viability Assays

The cell viability was determined using a 3-(4,5-dimethylthiazol-2-yl)-2,5-diphenyltetrazolium bromide (MTT) assay, which tests the normal metabolic status of the cells, based on the estimated mitochondrial activity. For the MTT assay, HepG2 and AML12 cells were seeded in 96-well culture plates at a concentration of 1.0 × 10^4^ cells/well and allowed to attach overnight. The cells were incubated with 0.5 mg/mL MTT at 37 °C for 1 h. The formazan granules generated by live cells were dissolved in dimethyl sulfoxide (DMSO) and the absorbance of the converted dye was measured at 540 nm using a MULTISKAN GO reader (Thermo Scientific, Waltham, MA, USA). The results are expressed as a percentage (%) of the vehicle-treated cell results.

### 2.3. Fluorescence-Activated Cell Sorting Analysis of Apoptosis

The numbers of apoptotic and live cells were determined by fluorescence-activated cell sorting (FACS) analysis using annexin V-FITC staining. The HepG2 cells were incubated with PHMG-P (Korea Institute of Toxicology, Jeongeup, Korea) for 24 h and subsequently harvested, trypsinized, washed with cold PBS, and resuspended in 1× binding buffer. The counted cells were stained with a propidium iodide (PI) and annexin V-FITC solution (Annexin V-FITC Apoptosis Detection Kit; BD Biosciences, Bedford, MA, USA) at room temperature for 15 min in the dark. The stained cells were analyzed by flow cytometry within 1 h. The apoptotic and live cells were analyzed using a Becton Dickinson FACSscan flow cytometer and BD FACSDiva software (BD Biosciences, San Jose, CA, USA).

### 2.4. FACS Analysis of Mitochondrial Membrane Potential

The HepG2 cells were incubated with PHMG-P for 16 h or 24 h. After treatment, the cells were incubated with 10 μM JC-1 dye in the culture medium for 30 min in the dark and were later collected by scraping. After washing in PBS, the cells were subjected to FACS analysis. Two excitation wavelengths, 527 nm (green) for the monomer form and 590 nm (red) for the JC-1 aggregate form, were used. When the cells have normal mitochondrial function, the mitochondrial membrane potential (∆ψ) is high and red fluorescence is predominant. However, when there is mitochondrial injury, ∆ψ is reduced, leading to an increase in green fluorescence. Thus, the quantitation of red and green fluorescent signals reflects mitochondrial damage. The change in ∆ψ was monitored using the Becton Dickinson FACSscan flow cytometer and BD FACSDiva software.

### 2.5. Luciferase Reporter Assay for ER Stress Response

The pNL [NlucP/ATF6-RE/Hygro] and pNL [NlucP/ATF4-RE/Hygro] (with the NanoLuc luciferase construct), and pGL4.54 [luc2/TK] (with Firefly luciferase construct) vectors were obtained from Promega (Madison, WI, USA). The HepG2 cells were plated overnight in 12-well plates and then transfected with the NanoLuc luciferase construct and pGL4.54 [luc2/TK] plasmid in the presence of Lipofectamine^®^ 3000 (Invitrogen, San Diego, CA, USA) for 24 h. After 4 h of treatment of PHMG-P, luciferase activity was measured by Nano-Glo^®^ Dual-Luciferase^®^ Reporter Assay System (Promega) according to the manufacturer’s instructions. The relative luciferase activities were calculated by normalizing the NanoLuc luciferase activity with that of Firefly luciferase.

### 2.6. Western Blotting Analysis

After PHMG-P treatment, the cells were harvested with cold PBS. The cells were lysed with an ice-cold ProEXTM CETi protein extract solution (Translab, Daejeon, Korea) and the total protein concentration was determined using a BCA reagent (Thermo Scientific, Sunnyvale, CA, USA). The protein extracts were denatured by boiling at 100 °C for 5 min in 2× Laemmli sample buffer (Bio-Rad, Hercules, CA, USA). Equal amounts of the total protein per sample were separated by SDS-PAGE and transferred onto nitrocellulose membranes (Bio-Rad). The membranes were blocked with Tris-buffered saline with 0.1% Tween-20 (TBS-T), containing 5% non-fat powdered milk, for 1 h at room temperature. The membranes were then washed with TBS-T buffer and incubated overnight at 4 °C with the following specific primary antibodies (dilution 1:2000 to 1:5000): anti-p53, anti-p21, anti-Bax, anti-Bcl-2, and anti-GAPDH (Santa Cruz Biotechnology, Dallas, TX, USA); anti-caspase-3, anti-PARP, anti-CHOP, and anti-p-JNK (Cell Signaling, Beverly, MA, USA); and anti-p-IRE (Abcam, Cambridge, UK). After washing with TBS-T, the membranes were incubated for 1 h with the appropriate horseradish peroxidase-conjugated secondary antibodies. The resulting antigen-antibody complexes were detected using an EZ-Western Lumi Pico detection kit (DOGEN, Seoul, Korea).

### 2.7. Caspase-3 Activity

The HepG2 cells were incubated with various concentrations of PHMG-P for 16 h or 24 h. Caspase-3 activity was then determined by fluorometrically measuring the substrate cleavage. To evaluate caspase-3 activity, the cell lysates were prepared after their respective treatment with PHMG-P. The assays were performed in 96-well microtiter plates by incubating 50 μL of the cell lysate in 100 μL of reaction buffer (0.5 M HEPES, 1 M NaCl, 1 M DTT) containing the caspase-3 substrate (Ac-DEVD-AMC) at 5 μM. The lysates were incubated at 37 °C for 4 h. Thereafter, the fluorescence was measured at 460 nm, after the excitation at 380 nm, using a Glomax microplate reader (Promega).

### 2.8. Real-Time Reverse Transcription-Polymerase Chain Reaction

The total RNA were isolated from the cells using a Direct-zol™ RNA kit (Zymo Research, Orange, CA, USA). The cDNA was synthesized using an iScript™ cDNA Synthesis System (Bio-Rad). Real-time PCR was performed using a SensiFAST SYBR qPCR mix (Bioline, London, UK), according to the manufacturer’s protocol. The gene expression values were normalized relative to 18S expression values. The primer sequences used in these experiments are listed in Table 1.

### 2.9. Statistical Analysis

All results, expressed as the mean ± SD, were analyzed using a two-tailed Student’s *t*-test or one-way analysis of variance (ANOVA), followed by the Newman-Keuls multiple comparisons test. The acceptable level of significance was set at *p* < 0.05.

## 3. Results

### 3.1. PHMG-P Cytotoxicity in Liver Cells

**Figure 1:**
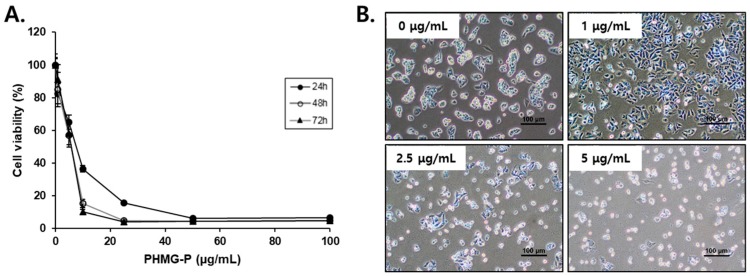
(**A**) The effect of polyhexamethyleneguanidine phosphate (PHMG-P) on HepG2 cell viability. The cells were treated with increasing concentrations of PHMG-P for 72 h, and then 3-(4,5-dimethylthiazol-2-yl)-2,5-diphenyltetrazolium bromide (MTT) assays were performed to measure the cell viability. The cell viability (%) is expressed as a percentage of the viability of vehicle-treated cells, and the data are presented from three independent experiments. Each value represents the mean ± SD. (**B**) The morphological changes in HepG2 cells after the treatment with PHMG-P for 24 h.

PHMG-P displayed significant cytotoxicity in HepG2 (Figure 1) and AML12 (Figure S1A) cells, as shown by the time- and concentration-dependent decrease in cell viability. The IC_50_ values obtained after 24, 48, and 72 h of PHMG-P incubation in HepG2 cells were 7.612, 5.822, and 5.840 μg/mL, respectively. In AML12 cells, the IC_50_ values after 24 and 48 h of PHMG-P incubation were 5.290 and 2.048 μg/mL, respectively. The cytotoxicity of PHMG-P in this study was in a similar range as that previously reported in A549, BEAS-2B, MRC-5, and THP-1 cells [2,3,20,21]. However, HepG2 and AML12 cells appear to be considerably more susceptible to PHMG-P than murine macrophage RAW264.7 cells, in which the IC_50_ values for 6 h and 24 h of exposure to PHMG-P were reported as 11.50 and 0.99 mg/mL, respectively [22].

### 3.2. Apoptosis Induced by PHMG-P in HepG2 Cells

**Figure 2:**
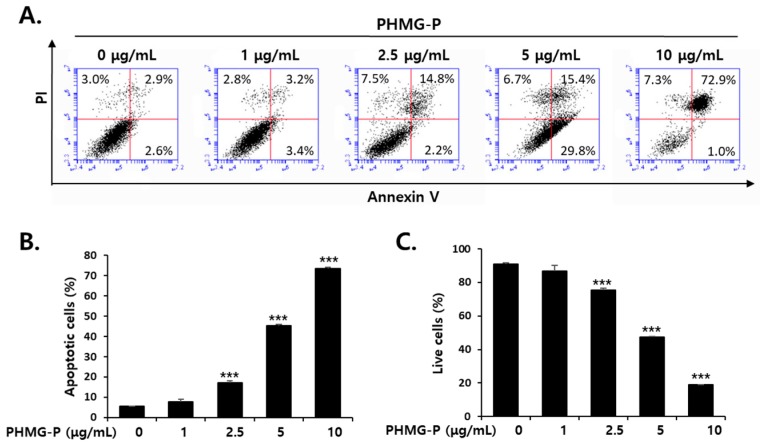
The induction of apoptosis in HepG2 cells treated with PHMG-P. (**A**) The cells were treated with increasing concentrations of PHMG-P for 16 h. Fluorescence-activated cell sorting (FACS) analysis of propidium iodide (PI) uptake and annexin V binding in non-permeabilized cells (lower left, live cells; lower right, early apoptotic cells; upper right, late apoptotic cells; upper left, necrotic cells). The quantification of (**B**) apoptotic cells and (**C**) live cells from three independent experiments. (*** *p* < 0.001, compared with vehicle-treated cells).

The cell surface exposure of membrane phosphatidylserine (PS), a classical feature of apoptosis, is a signal for the recognition and removal of apoptotic cells by phagocytes [23,24]. To determine whether apoptotic or necrotic cell death was taking place, a FACS analysis was performed using annexin V, which specifically and strongly binds to cell surface PS, and PI, which cannot penetrate the intact membrane of live or early apoptotic cells [25]. The exposure of HepG2 cells to 1, 2.5, 5, or 10 μg/mL PHMG-P for 24 h resulted in the concentration-dependent induction of apoptosis (Figure 2). PMHG-P at 2.5–5 μg/mL caused both apoptosis and necrosis (Figure 2A). However, 72.9% of the cells treated with 10 μg/mL PHMG-P showed features of late apoptosis, whereas only 7.3% of these cells died via necrosis (Figure 2A,B), suggesting that apoptosis may be the major pathway of PHMG-P-induced cell death in HepG2 cells.

**Figure 3:**
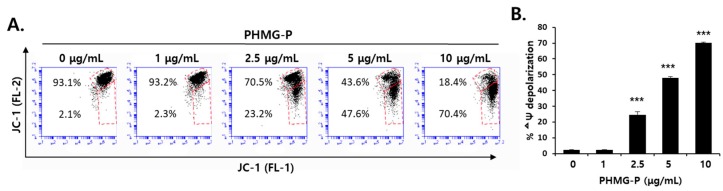
The induction of mitochondrial dysfunction in HepG2 cells treated with PHMG-P. (**A**) The cells were treated with increasing concentrations of PHMG-P for 16 h prior to FACS analysis of JC-1 staining. (**B**) Quantification of mitochondrial membrane potential (ΔΨ) from three independent experiments. Each value represents the mean ± SD. (*** p < 0.001, compared with untreated cells).

The mitochondrial membrane potential analysis using the fluorescent dye JC-1, indicated that PHMG-P exposure led to significant depolarization of the mitochondria (Figure 3). In normal cells, JC-1 enters energized the mitochondria that have high membrane potential and form aggregates that exhibit red fluorescence [26]. However, in unhealthy or apoptotic cells with low mitochondrial membrane potential, JC-1 remains in the monomeric form, which shows green fluorescence [26]. The cells exposed to PHMG-P at 2.5, 5, or 10 μg/mL for 24 h showed mitochondrial membrane depolarization at a rate of 23.2, 47.6, and 70.4%, respectively (Figure 3).

**Figure 4:**
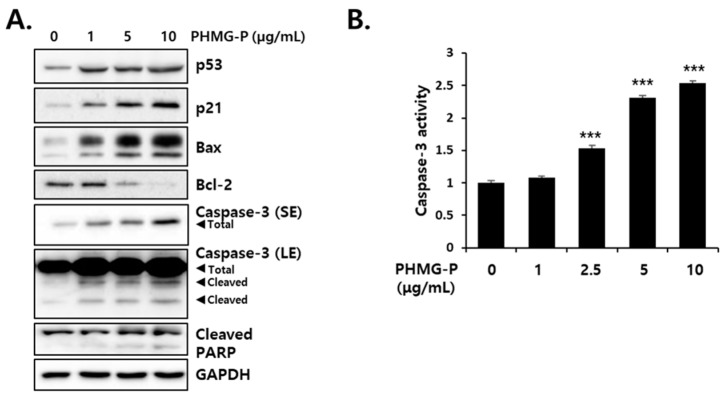
The effect of PHMG-P on the levels of apoptosis-related proteins and caspase activity in HepG2 cells. (**A**) The cells were treated with PHMG-P at 1, 5 and 10 μg/mL for 16 h. Western blotting analysis was performed using specific antibodies against the indicated proteins. GAPDH was used as a loading control. (**B**) Cells were treated with PHMG for 6 h and caspase-3 activity was determined using a fluorometric substrate. The results are presented as the mean ± SD of triplicate experiments. SE: short exposure, LE: long exposure. (*** *p* < 0.001 compared with vehicle-treated cells).

PHMG-P also activated the apoptotic signaling pathway in a concentration-dependent manner. The protein levels of p53, which is known to initiate apoptosis, and the cell cycle inhibitor, p21, which is activated by p53, increased in liver cells treated with PHMG-P (Figure 4). Bax, a pro-apoptotic protein in the Bcl-2 family, was detected at high levels in PHMG-P-treated cells, whereas the levels of the anti-apoptotic protein, Bcl-2, decreased after PHMG-P treatment (Figure 4). Caspase-3, a critical mediator of apoptosis, catalyzes the cleavage of poly (ADP-ribose) polymerase (PARP) and thus, cleaved PARP is a biomarker of caspase activity in apoptotic cells [27,28]. The protein levels of total and cleaved caspase-3 (Figure 4A) and caspase-3 activity (Figure 4B) increased by 2.5–10 μg/mL PHMG-P treatment. The PARP fragmentation was also induced by 24 h of treatment with PHMG-P (Figure 4A). These results clearly demonstrate the induction of apoptosis by PHMG-P in liver cells.

### 3.3. ER Stress Induced by PHMG-P in HepG2 Cells

**Figure 5:**
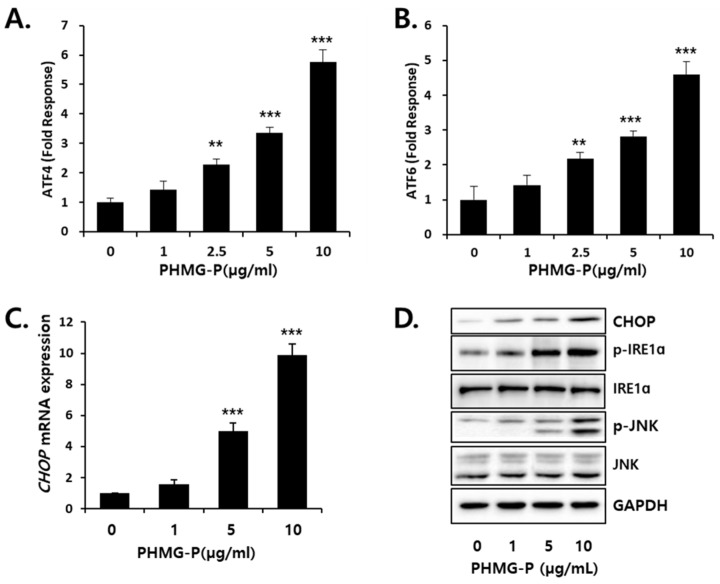
The induction of ER stress in PHMG-P-treated HepG2 cells. The cells were exposed to indicated concentrations of PHMG-P for 4 h, and ER stress response was determined by (**A**) ATF-4- and (**B**) ATF-6-luciferase activity. PHMG-P upregulated the expression of (**C**) CHOP mRNA and (**D**) ER stress-related proteins in HepG2 cells. The cells were treated with PHMG-P at 1, 5, and 10 μg/mL for 16 h. The results are presented as the mean ± SD of triplicate experiments. (** *p* < 0.01, *** *p* < 0.001 compared with vehicle-treated cells).

ATF4 and ATF6 are known to be the primary transcriptional regulators of several ER-chaperones. This study determined whether PHMG-P can induce ATF4 (Figure 5A) and ATF6 (Figure 5B) transcriptional activity using the reporter vectors containing ATF4- or ATF6-response element. The transcriptional activities of both ATF4 and ATF6 significantly increased from 2.5 μg/mL PHMG. The mRNA (Figure 5C) and protein levels (Figure 5D) of C/EBP homologous protein (CHOP), a marker of ER stress, were elevated by PHMG-P treatment. The inositol-requiring enzyme (IRE), a UPR sensor, is activated by phosphorylation during ER stress conditions [29,30]. The activated IRE recruits the tumor necrosis factor receptor-associated factor 2 (TRAF2), resulting in the phosphorylation of c-Jun N-terminal kinase (JNK), which upregulates pro-apoptotic genes [31]. The protein levels of p-IRE and p-JNK increased in the cells exposed to PHMG-P (Figure 5D).

### 3.4. Inhibition of PHMG-P-Induced ER Stress by TUDCA

**Figure 6:**
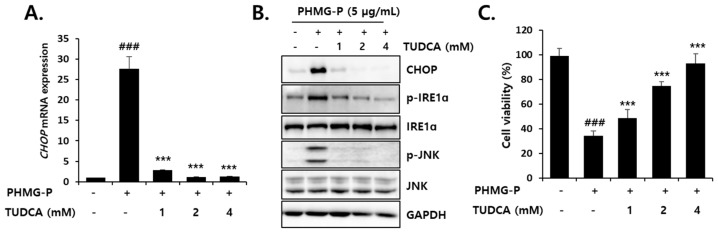
The preventive effect of ER stress inhibitor, TUDCA, on PHMG-P-induced cytotoxicity in HepG2 cells. The cells were pretreated with TUDCA for 2 h and then treated with 5 µg/mL PHMG-P for 24 h. The expression of (**A**) CHOP mRNA and (**B**) ER stress-related proteins were determined using real-time PCR and western blotting, respectively. (**C**) The MTT assays were performed to measure cell viability. The cell viability (%) was expressed as a percentage of the viability of untreated cells. Each value represents the mean ± SD of triplicate experiments. (### *p* < 0.001, significant versus vehicle-treated; *** *p* < 0.001, significant versus PHMG alone).

To clarify the relationship between ER stress and the cytotoxicity of PHMG-P, the cells were treated with TUDCA, before and during PHMG-P (5 µg/mL) exposure. The PHMG-P-induced elevation of CHOP, p-IRE, and p-JNK levels were significantly inhibited by 1–4 mM TUDCA (Figure 6). The decreased viability of the cells exposed to PHMG-P alone was restored by TUDCA in a dose-dependent manner and 4 mM TUDCA completely prevented PHMG-P cytotoxicity (Figure 6C, Figure S1B). These results suggest that ER stress may be a major cause of PHMG-P-induced cell death.

### 3.5. Inhibition of PHMG-P-Induced Apoptosis by TUDCA

**Figure 7:**
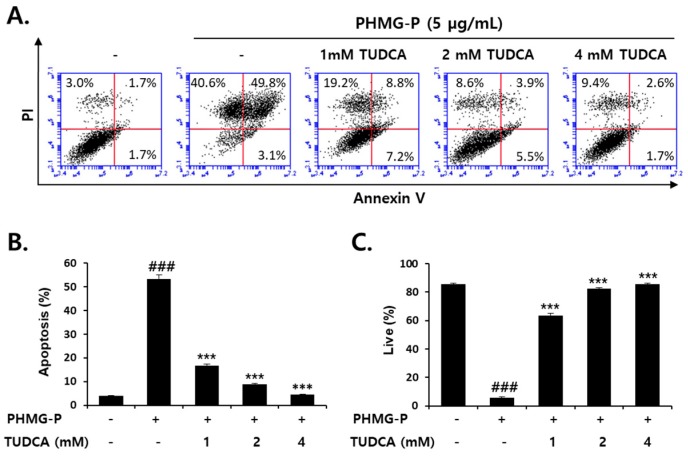
The inhibition of PHMG-P-induced apoptotic cell death by the ER stress inhibitor, TUDCA. (**A**) The cells were pretreated with TUDCA and then treated with 5 µg/mL PHMG-P for 24 h. The FACS analysis of propidium iodide uptake and annexin V binding in non-permeabilized cells (lower left, live cells; lower right, early apoptotic cells; upper right, late apoptotic cells; upper left, necrotic cells). (**B**) The quantification of apoptotic cells is presented from three independent experiments. (**C**) The quantification of live cells is presented from three independent experiments. (### *p* < 0.001, significant versus control; *** *p* < 0.001, significant versus PHMG alone).

**Figure 8:**
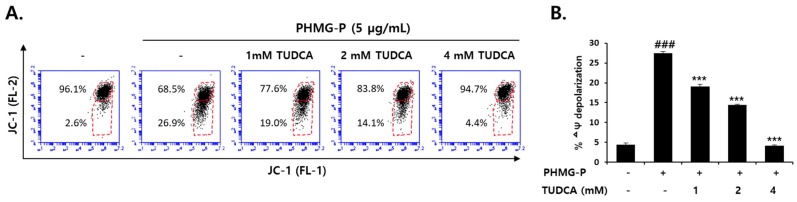
The inhibition of PHMG-P-induced mitochondrial dysfunction by the ER stress inhibitor, TUDCA. (**A**) The cells were pretreated with TUDCA and then treated with 5 µg/mL PHMG-P for 24 h prior to FACS analysis of JC-1 staining. (**B**) The quantification of mitochondrial membrane potential (ΔΨ) is presented from three independent experiments. Each value represents the mean ± SD. (### *p* < 0.001, significant versus control; *** *p* < 0.001, significant versus PHMG alone).

**Figure 9:**
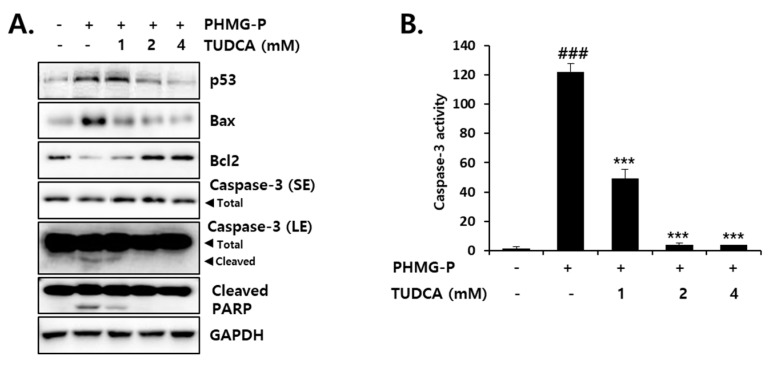
The inhibition of PHMG-P-induced apoptosis-related proteins and caspase-3 activity by the ER stress inhibitor, TUDCA. The cells were pretreated with TUDCA and then treated with 5 µg/mL PHMG-P for 24 h. (**A**) Western blotting was performed to determine apoptosis-related protein levels. GAPDH was used as an internal control. (**B**) The cell lysates were prepared and caspase activity was then measured using a fluorometric substrate. The results are presented as the mean ± SD of triplicate experiments. SE: short exposure, LE: long exposure. (### *p* < 0.001, significant versus control; *** *p* < 0.001, significant versus PHMG alone).

Apoptotic cell death induced by PHMG-P (5 μg/mL) was also significantly inhibited by TUDCA in HepG2 cells (Figure 7). TUDCA (1–4 mM) markedly reduced the ratio of apoptotic cells that were increased by PHMG-P (Figure 7A,B). The number of necrotic cells was also decreased by TUDCA (Figure 7A). However, the inhibition of apoptosis (52.9% to 4.3% of cells) was more evident than the inhibition of necrosis (40.6% to 9.4%) in TUDCA-treated cells (Figure 7A). These results suggested that PHMG-P-induced ER stress was more closely related to apoptotic cell death. Mitochondrial depolarization induced by PHMG-P (5 μg/mL) was restored by TUDCA in a concentration-dependent manner (Figure 8). The activation of the apoptotic signaling cascade by PHMG-P was also attenuated by the TUDCA treatment (Figure 9). TUDCA reduced p53, Bax, cleaved caspase-3, and cleaved PARP protein levels but increased the levels of the anti-apoptotic protein, Bcl-2, in PHMG-P-treated HepG2 cells (Figure 9A). The activation of caspase-3 by PHMG-P was also markedly inhibited by TUDCA treatment (Figure 9B). These results indicate that ER stress may be an important mechanism of PHMG-P-induced hepatic cell apoptosis.

## 4. Discussion

According to both epidemiological and experimental studies, it is clear that exposure to humidifiers disinfected with PHMG-P induces severe lung injury, mediated by inflammatory and fibrotic responses and apoptosis. Recently, this study observed that the relative distribution and retention times of both inhaled and instilled PHMG-P are higher in the liver than in other organs, raising the possibility of harmful effects on the liver. In the present study, significant levels of apoptosis were induced in liver cells due to the cytotoxicity of PHMG-P (Figure 1 and Figure 2). Previously, a similar dose range of PHMG-P (9.6–16.0 µg/mL) was shown to induce apoptotic cell death in cultured human bronchial cells [2]. Therefore, these results suggested that apoptosis may be the major pathway of cell death induced by PHMG-P in liver cells and pulmonary cells.

Various cellular organelles are involved in apoptosis, but mitochondria play a prominent role in the initiation of this cell death process [32,33]. B-cell lymphoma 2 (Bcl-2) family proteins in the outer mitochondrial membrane regulate mitochondria-mediated apoptosis. These proteins are divided into three groups: Anti-apoptotic (Bcl-2, Bcl-xL, Bcl-W, Mcl-1, etc.), pro-apoptotic (Bax and Bak), and apoptosis initiator (Bad, Bid, Bim, Puma, and Noxa) proteins [34,35]. The activation of apoptotic signals by PHMG has been well defined in pulmonary cells. The human bronchial cells incubated with PHMG-P (3.2–16.0 µg/mL) for 24 h showed dose-dependent increases in pro-apoptotic signals, such as Bax, Apaf-1, cytochrome c, cleaved caspase-3, and cleaved PARP, but a decrease in levels of the anti-apoptotic protein, Bcl-2 [2]. Moreover, the elevated protein levels of p53, Bax, Bak, and Bid have been observed in human alveolar (A549) cells treated with 2–10 µg/mL PHMG-H for 24 h [20]. In the present study, PHMG-P treatment of HepG2 cells resulted in a dose-dependent decrease in mitochondrial membrane potential (ΔΨ), which is a critical driving force for ATP synthesis (Figure 3). The PHMG-P-induced increase in Bax protein and a decrease in Bcl-2 protein levels (Figure 4) may be the causes of mitochondrial depolarization, most likely due to pore formation. The significant increases in the quantity and activity of cleaved caspase-3 protein (Figure 4) suggest that PHMG-P-induced apoptosis is mediated in a caspase-dependent manner.

The ER is abundant in liver tissue, with microsomal structures to facilitate the synthesis of various secretory proteins, such as albumin, fibrinogen, clotting factors, and lipoproteins. The eukaryotic ER is a primary site for protein folding, which is accomplished by the coordination among ER-resident proteins, including foldases, calreticulin, protein disulfide isomerase, and chaperones [36,37,38]. This is because the ER stores Ca^2+^, and high levels of Ca^2+^ are required for chaperone function and protein folding reactions [30,38,39]. The ER stress-inducing agents, thapsigargin and tunicamycin, have been shown to cause the release of Ca^2+^ from the ER into the cytoplasm, and this free Ca^2+^ is taken up by mitochondria [40]. The mitochondrial accumulation of Ca^2+^ then leads to membrane depolarization and cytochrome c release, which initiates apoptosis [38]. The presence of intense and sustained ER stress eventually induces apoptosis [29,36,38,41]. Thus, the liver is particularly vulnerable to ER stress, which is recognized as a significant mediator of various liver diseases [42].

When ER stress occurs, glucose-regulated protein 78 (GRP78), a chaperone in the ER lumen, preferentially associates with unfolded proteins and dissociates from the three sensors being activated [43]. IRE acts as a Ser/Thr protein kinase and endoribonuclease [38]. IRE, once dissociated from GRP78, is activated by auto-phosphorylation and then degrades RNA to reduce protein synthesis. This process is known as regulated IRE-dependent mRNA decay (RIDD) [44]. The transcription factor X box-binding protein 1 (XBP1) is the main target of the ribonuclease activity of IRE [29]. The activated IRE removes a 26-base intron from the mRNA of unspliced XBP1 (uXBP1), and the translated, spliced form of XBP1 (sXBP1) then binds to ER stress response elements (ERSEs) to activate the transcription of genes involved in ER biogenesis, chaperone synthesis, and ER-associated degradation [45,46]. The activated PERK is known to phosphorylate and inhibit the eukaryotic translation-initiation factor-2α (eIF2α), which rapidly reduces the general initiation of mRNA translation, to limit the protein-folding load [47]. However, phosphorylated eIF2α translates several specific mRNAs, such as ATF4, which then induce CHOP [47,48]. The activated ATF6 translocates to the Golgi apparatus to be cleaved, after which it migrates into the nucleus to activate the transcription of chaperones, protein disulfide isomerase, XBP1, and CHOP [41,49,50]. In the present study, the increase in activated IRE seen after PHMG-P treatment (Figure 5) indicated the dissociation of this protein from GRP78 in the ER. CHOP is a well-known marker of ER stress, as this transcription factor is induced via the PERK/eIF2a/ATF4 [51] and ATF6 [52] pathways. Thus, the alterations in phospho-IRE and CHOP levels in HepG2 cells is clear evidence of PHMG-P-induced ER stress.

The Bcl-2 family proteins, which also localize to the ER membrane, are known to be involved in the release of Ca^2+^. The mouse embryonic fibroblasts deficient in Bax and Bak show a diminished release of ER Ca^2+^, whereas, the overexpression of Bax causes the ER release and mitochondrial accumulation of Ca^2+^ [53]. In ER stress conditions, an increase in Bax/Bak levels and their accumulation in the ER and mitochondrial membranes have been observed [40,54]. The activation of IRE is thought to be important for Bax/Bak-mediated apoptotic signals. The activated IRE recruits TRAF2 and forms a complex with apoptosis signal-regulating kinase 1 [29,38,41]. This complex activates JNK, which then phosphorylates Bcl-2 in the ER membrane to dissociate it from Bax and Bak [55]. As a result, the oligomerization of Bax/Bak on the ER surface leads to Ca^2+^ release [41]. Moreover, p-JNK can activate p53 [41], which is known to induce Bax [35]. In the present study, PHMG-P-induced ER stress appeared to be closely associated with apoptotic cell death. The activation of IRE/JNK and the induction of Bax suggest that the apoptosis induced by PHMG-P may be caused by the disruption of calcium homeostasis resulting from ER stress. CHOP has been reported to inhibit Bcl-2 expression and sensitize cells to ER stress [56]. Thus, the reduction in Bcl-2 levels by PHMG-P may be due to the activation of the PERK/eIF2α/ATF4/CHOP pathways.

To confirm the involvement of ER stress in PHMG-P-induced apoptosis, the cells were treated with TUDCA, a taurine conjugate of ursodeoxycholic acid that is known to prevent ER stress [57,58,59]. TUDCA inhibits the increase in GRP78, ATF4, and CHOP levels and the sXBP1/uXBP1 ratio in cultured liver cells treated with tunicamycin [57]. The reduction in ER stress by TUDCA results in the inhibition of apoptosis [58,59,60,61]. Thapsigargin-induced apoptosis that occurs via mitochondrial depolarization, cytochrome c release, caspase-3 activation, and Ca^2+^ efflux from the ER, was significantly decreased by TUDCA in HepG2 cells [58]. The UPR-stabilizing effect of TUDCA is considered to be due to its binding to the hydrophobic regions of proteins, which prevents protein aggregation and improves protein folding capacity [62,63]. Thus, this bile acid is known as a chemical chaperone. In the present study, TUDCA treatment significantly inhibited ER stress and PHMG-P cytotoxicity in liver cells (Figure 6). A marked reduction in apoptotic cell death (Figure 7) via the restoration of mitochondrial membrane potential (Figure 8) and the normalization of apoptotic protein levels (Figure 9) by TUDCA strongly suggested that ER stress is a critical upstream signal for the induction of apoptosis in cells exposed to PHMG-P.

In summary, PHMG-P showed potent cytotoxicity, and mitochondria-mediated apoptosis may be the major pathway of PHMG-P-induced liver cell death. Of note, this study described, for the first time to the authors’ knowledge, that PHMG-P induced significant levels of ER stress in liver cells. Moreover, it was shown that ER stress may be an important mechanism of PHMG-P-induced apoptosis. Thus, it can be concluded that PHMG-P induced ER stress-mediated apoptosis, leading to hepatotoxicity. Further studies are required to clarify the exact physical and/or biochemical actions of PHMG on the hepatic ER.

## Figures and Tables

**Table 1:** List of primers used for real-time reverse transcription-polymerase chain reaction (RT-PCR).

Genes	Primer Sequences	
*CHOP*	F: CAGAACCAGCAGAGGTCACA	R: AGCTGTGCCACTTTCCTTTC
*18S*	F: CAGCCACCCGAGATTGAGCA	R: TAGTAGCGACGGGCGGTGTG

## Supplementary Materials

The following are available online at https://www.mdpi.com/2073-4409/8/9/1023/s1, Figure S1: Effect of PHMG-P on cell viability and preventive effect of TUDCA on PHMG-P-induced cytotoxicity in AML12 cells.
