# Supplementary material for: Polyhexamethyleneguanidine Phosphate-Induced Cytotoxicity in Liver Cells Is Alleviated by Tauroursodeoxycholic Acid (TUDCA) via a Reduction in Endoplasmic Reticulum Stress
Source: Cells. 2019 Sep 3;8(9):1023. doi: 10.3390/cells8091023 (PMC6770470; doi:10.3390/cells8091023)
Supplement: Supplementary file 1 [file cells-08-01023-s001.pdf]

cells

MDPI

Supplementary figure

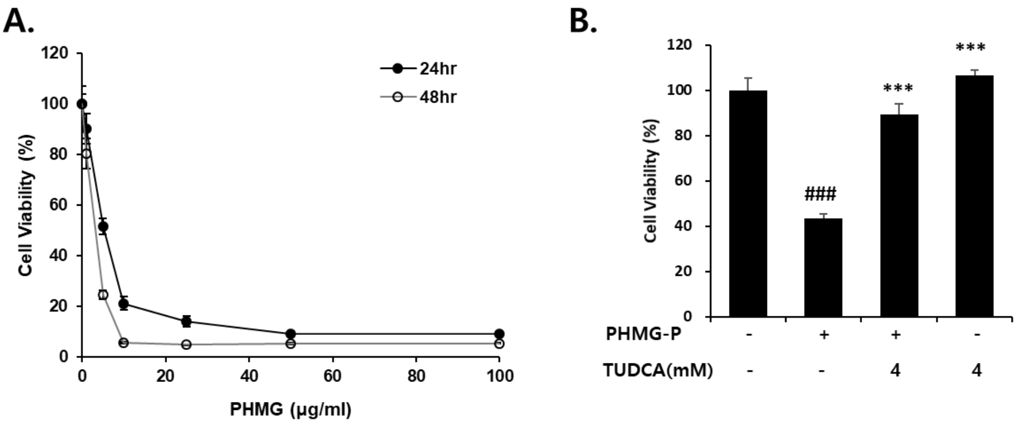

Figure S1. Effect of PHMG-P on cell viability and preventive effect of TUDCA on PHMG-P-induced cytotoxicity in AML12 cells. (**A**) Cells were treated with increasing concentrations of PHMG-P for 48 h, and then MTT assays were performed to measure cell viability. Cell viability (%) is expressed as a percentage of the viability of vehicle-treated cells, and data are presented from three independent experiments. Each value represents the mean ± SD. (**B**) Cells were pretreated with TUDCA for 2 h and then treated with 5 μg/mL PHMG-P for 24 h. MTT assay was performed to measure cell viability. Cell viability (%) was expressed as a percentage of the viability of untreated cells. Each value represents the mean ± SD of triplicate experiments. (### $p < 0.001$, significant versus vehicle-treated; *** $p < 0.001$, significant versus PHMG alone)
